# Antibiotic artificial bone implantation and external fixation for the treatment of infection after intramedullary nail fixation: a retrospective study of 33 cases

**DOI:** 10.1186/s12891-022-05161-8

**Published:** 2022-03-05

**Authors:** Haotian Hua, Lei Zhang, Zairan Guo, Wenlong Zhong, Jiangfei Chen, Shilin Wang, Jiangang Guo, Xinwei Wang

**Affiliations:** 1 Research and Treatment Center of Bone and Joint Infections, Luoyang Orthopedic-Traumatological Hospital of Henan Province (Henan Provincial Orthopedic Hospital), Luoyang, China; 2 Henan University of Traditional Chinese Medicine, Zhengzhou, China

**Keywords:** Antibiotic artificial bone, External fixation, Intramedullary nail, Infection

## Abstract

**Objective:**

To explore the clinical effect of antibiotic artificial bone implantation and external fixation in the treatment of infection after intramedullary nail fixation.

**Methods:**

We retrospectively reviewed the clinical data of patients with infection after intramedullary nail fixation treated from March 2010 to August 2020. There were 27 males and 6 female, aged from 12 to 67 years (average 42.27 years), 18 cases on the left side and 15 cases on the right side. Among them, 20 cases were open fractures with initial injury and 13 cases were closed fractures. All patients were treated with intramedullary nail removal, local debridement, antibiotic artificial bone implantation and external fixation. Because of bone defects, 19 patients underwent secondary autologous cancellous bone grafting after infection control. Postoperative wound healing, related inflammatory indicators, fixation time, and bone healing time were recorded and followed up.

**Results:**

The 33 patients were followed up with period of 10 ~ 98 months (average 62.7 months). One patients failed to control the infection effectively after treatment, so received antibiotics artificial bone implantation again. Two patients also received antibiotic artificial bone implants again due to the recurrence of the infection. After treatment, infection was controlled and the fracture healed well. One patient received vacuum sealing drainage (VSD) due to persistent postoperative exudation, and five patients were also cured successfully after continuous dressing. Two patients had sinus tract after surgery, and the wound was cured by continuous dressing change. Nineteen patients received autogenous iliac bone grafts for healing due to bone defects ranging from 3 to 6.5 cm (average 4.15 cm) after infection control. The external fixation time of 33 patients ranged from 4 to 16 months (average 7.79 months), the bone healing time ranged from 4 to 13 months (average 6.67 months), and the related inflammatory indexes returned to normal within 2–8 weeks (average 4.48 weeks).

**Conclusion:**

Antibiotic artificial bone implantation and external fixation is an effective method for the treatment of infection after intramedullary nail fixation.

**Supplementary Information:**

The online version contains supplementary material available at 10.1186/s12891-022-05161-8.

## Background

Intramedullary nail fixation has become the gold standard for the treatment of long bone fractures due to its superior mechanical properties and minimally invasive characteristics [1]. With the clinical application increasing, infections after intramedullary nail fixation occur from time to time, and the incidence of infection after intramedullary nail fixation has been reported to reach 0.9% ~ 3.8% [2]. In view of the characteristics of intramedullary nail fixation, bacteria will spread to the entire medullary cavity along the intramedullary nail once infection occurs. It may develop into chronic osteomyelitis and affect fracture healing seriously, leading to dysfunction of the affected limb if not treated in time [3]. Infection after intramedullary nail fixation is one of the intractable problems in orthopedic clinics, and an unified standard treatment plan has not yet been formed. Previous scholars reported the removal of intramedullary nails, local debridement and irrigation for the treatment of infection after intramedullary nails, but this method is difficult to remove residual bacteria in the medullary cavity completely, and the recurrence rate is relatively high [4]. In order to reduce the recurrence of infection, some scholars implanted bone cement beads mixed with antibiotics to eliminate residual bacteria after removal of intramedullary nails and local debridement [5]. In view of chain beads cannot provide good mechanical support, some scholars implanted bone cement rods mixed with antibiotics into the medullary cavity instead of chain beads to control infection and strengthen the stability of the fractures [6]. However, this material cannot be absorbed after being implanted in human body, and must be taken out by second operation. Moreover, the release level of antibiotic is unstable extremely, which will enter a low-level release cycle soon after the early explosive release [7]. Not only can it not kill bacteria, but also become a carrier for bacterial colonization as a foreign object in the body [8]. Antibiotic-impregnated calcium sulfate, as a new topical antibiotic delivery system, has been used in clinical practice widely [9, 10]. Existing reports have demonstrated its effectiveness in the treatment of chronic osteomyelitis [11–13]. According to literature reports and our experience, on the basis of removing the intramedullary nail and reaming, we used antibiotic artificial bone implantation and external fixation to control infection after intramedullary nail fixation. The purpose of this study is to evaluate the effectiveness of this method.

## Methods

In this study, the clinical data of 33 patients diagnosed with infection after intramedullary nail fixation and treated at our departent from March 2010 to August 2020 were retropectively analyzed. The study was approved by the Luoyang Orthopedic-traumatological Hospital’s ethical review committee (KY2018-001–01). Written informed consent was obtained from all patients to use their clinical data for the clinical research.

The inclusion criteria were as follows: (1) Infection after intramedullary nail fixation; (2) Patients were treated with intramedullary nail removal, local debridement, antibiotic artificial bone implantation and external fixation; (3) Retrospective study. The exclusion criteria were as follows: (1) Patients with severe liver and kidney insufficiency, cardiovascular and cerebrovascular diseases, diabetes and other medical diseases that affect the treatment effect; (2) Patients with severe osteoporosis; (3) Patients with intra-articular fracture. The diagnosis of infection after intramedullary nail fixation is based on the expert consensus,which comes from Association for the Study of Internal Fixation [14]. The contents include: (1) Patients with fistula and sinus that connected with implant or bone tissue; (2) During the operation, pus was found around the implants; (3) The bacterial culture of the suspected infected tissue was positive during the operation; (4) Histopathological examination confirmed the presence of pathogenic microorganisms in suspected infected tissues. Infection after intramedullary nail fixation can be diagnosed if one of the above conditions was met.

From March 2010 to August 2020, a total of 33 patients who met the inclusion criteria were included in the analysis. There were 27 males and 6 females, aged from 12 to 67 years (average 42.27 ± 14.80 years), 18 cases on the left side and 15 cases on the right side. Among them, 20 cases were open fractures with initial injury and 13 cases were closed fractures. All fractures were due to some form of trauma (traffic trauma in 21 cases, falling injury in 9 cases and heavy pound injury in 3 cases). Preoperative radiographs showed that 6 patients had healed fractures and 27 patients had unhealed fractures. The interval between the infection and the first operation from half a month to 12 months(average 3.53 ± 2.73 months). Routine examinations were performed after admission. The average preoperative white blood cell (WBC) was (9.31 ± 2.03) × 10^9^/L, the average preoperative C-reactive protein (CRP) was (34.46 ± 33.8) mg/L, and the preoperative erythrocyte sedimentation rate (ESR) was (34.85 ± 26.1) mm/ h. The basic information of patients were shown in Table 1.

**Table 1:** Patients demographics

Variables	
No.of cases	33
Sex(Male/Female)	27/6
Mean age(years)	42.27 ± 14.80
Location	
Femure	14
Tibia	19
Initial trauma	
Traffic trauma	21
Falling injury	9
Heavy pound injury	3
Open fracture	20
Closed fracture	13
The interval between the infection and the first operation(months)	3.53 ± 2.73
Fracture healed or not	6/27
Inflammatory indicators before surgery	
WBC	(9.31 ± 2.03) × 10^9^
CRP	34.46 ± 33.8
ESR	34.85 ± 26.1

Data shown as mean ± standard deviation. *P* < 0.05, significant difference

*WBC* white blood cell; *CRP* C-reactive protein; *ESR* erythrocyte sedimentation rate

### Surgical procedures

Preoperative treatment: Twenty-six patients with sinus tracts received bacterial culture of secretions. The results showed that: 16 cases of Staphylococcus aureus, 4 cases of Pseudomonas aeruginosa, 1 case of Enterobacter cloacae, 5 cases of negative. We selected sensitive antibiotics for antibacterial treatment based on the culture results, and if there is no sinus or the result is negative, antibiotics were used empirically. All patients received radiographs of the affected limbs to assess the healing of the fractures, clarify the bone defect and the scope of infection before the operation. In the present study, all the surgical procedures were performed by the same surgeon.

Operation method: After anesthesia, the patient was placed in a supine position, and the surgical area is disinfected routinely. The intramedullary nail was removed, and the inflammatory tissue, scars, pus, dead bones in the nail area and the fractured end were removed completely. Finally, the grinding drill was used to polish unitl the “chili sign” appeared [15]. The soft drill was used to ream the medulla and facilitate the removal of inflammatory tissue in the medullary cavity, and then send the necrotic or infected tissue removed from the medullary to bacterial culture. The standard for reaming is 1 ~ 2 mm larger than the original intramedullary diameter. when the fresh cancellous bone mud appeared in the groove of the soft drill bit, a long nozzle water gun was used to flush the medullary cavity along the opening of the intramedullary nail and the broken end of the fracture with no less than 6L of washing water [3]. After rinsed repeatedly with normal saline and hydrogen peroxide, infection area was soaked by iodophor solution for 10 mintues. Then replaced surgical drapes and gloves. The antibiotic artificial bone was prepared with a ratio of vancomycin 0.5 g + gentamicin 2 ml + calcium sulfate bone powder 5 ml (Biocomposites Ltd, England), and the mixed paste was implanted evenly into the mold to form the 4 mm × 3 mm balls. The prepared vancomycin calcium sulfate ball was placed and dried for 15 min, and then filled into the medullary cavity evenly. Unilateral external fixator was used for fixation. Finally, placed the drainage tube, sutured the wound in layers, and covered the wound with a sterile dressing. In consideration of the patient’s economic situation and the possible side effects of calcium sulfate bone powder, the total amount of calcium sulfate bone meal used by each patient did not exceed 50 ml.

Postoperative treatment: routine anticoagulant and analgesic therapy were performed after surgery. According to the results of preoperative bacterial culture, sensitive antibiotics were used for intravenous infusion and then switched to oral antibiotics for 4 weeks. If the bacterial culture results of the infected tissue taken out during the operation were inconsistent with the preoperative culture results, antibiotics should be adjusted in time. Surgical dressing change was performed regularly, wound healing and drainage tube were observed. Muscle exercises and related joint exercises are encouraged. Blood routine, C-reactive protein (CRP), erythrocyte sedimentation rate (ESR) were checked to understand the control of inflammation every two weeks after surgery. Radiographs were reviewed regularly to observe the healing of the fracture. Because of bone defects, 19 patients underwent autologous iliac bone graft surgery after infection control.

### Result evaluation

At present, there is no exact diagnosis and treatment evaluation standard for postoperative infection of intramedullary nails. We consulted relevant literature and evaluated the efficacy according to whether the fracture healing and infection control. Infection control includes: Disappearance of the patient’s systemic inflammatory symptoms. Wound healing, no local redness, no swelling and pain. CRP, ESR, and WBC indicators return to normal. Infection recurrence includes: Patients still has fever. Local redness, swelling, pain, sinus and poor wound healing. Abnormal CRP, ESR, and WBC indicators.

### Statistical analysis

IBM SPSS 21.0 software (SPSS Inc, USA) was used for statistical processing. Quantitative data was presented as mean ± standard deviation.

## Results

**Fig. 1:**
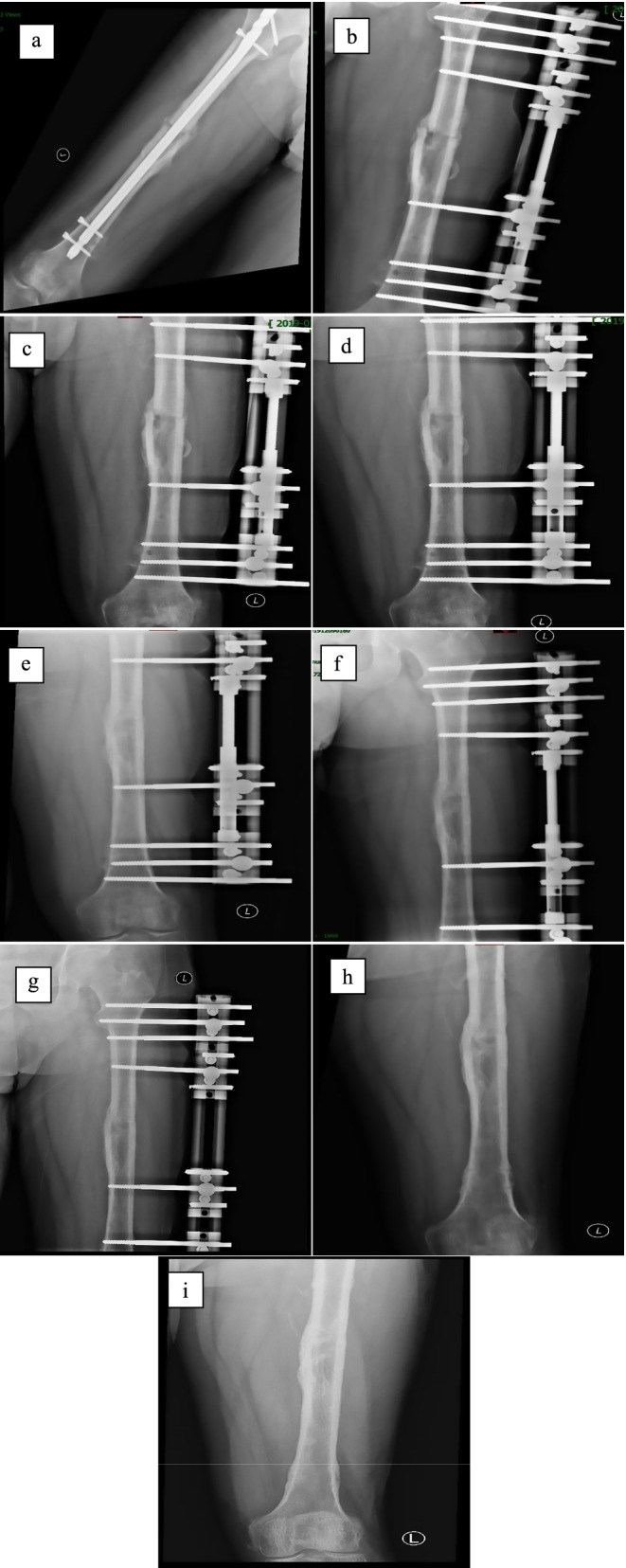
Typical case. A 37-year-old male patient with fracture of the femur due to traffic injury. Two months after intramedullary nail fixation, the patient developed swelling, ulceration, and purulent discharge on the medial femur. **a** preoperative radiographs showed intramedullary nail fixation of the femoral fracture. **b** one week after operation, radiographs showed that the intramedullary nail had been removed, the upper femoral medullary cavity was filled with artificial bone particles, and the external fixation was performed. **c** forty-five days after operation, bone callus can be seen at the fracture end, artificial bone particles were absorbed. **d** four months after operation, the callus grew well and the artificial bone particles were absorbed completely. **e** six months after operatian, the callus grew well and the external fixation was performed. **f** seven months after operation, the callus grew well and the external fixation was performed. **g** eight months after operation, the callus grew well and the extermal fixation was performed. **h** eleven months after operation, the fracture was healed, and the external fixator was removed. **i** sixteen months after operation, radiographs showed that the fracture healed well

The operations of all patients were completed successfully. The operation time was 110 ~ 240 min (average 158.49 ± 23.3 min), and intraoperative blood loss was 200 ~ 1000 ml (average 490.91 ± 298.3 ml). Thirty-three patients were followed up for 10 ~ 98 months after surgery (average 62.7 months). Because of poor infection control, one patients underwent debridement and artificial bone implantation again. Two patients also received antibiotic artificial bone implants again due to the recurrence of the infection.. After treatment, infection was controlled and the fracture healed well. Because of persistent postoperative exudation, one patient received vacuum sealing drainage (VSD), five patients were also cured successfully after continuous dressing. Because of the sinus left after the operation, two patients received continuous dressing, and the sinus healed well. After the first-stage operation, 19 patients received second-stage autologous iliac bone grafting due to residual bone defects. The bone defect ranged from 3 to 6.5 cm (average 4.15 ± 0.82 cm). The external fixation time was 4 ~ 16 months (average 7.79 ± 2.81 months). The bone healing time was 4 ~ 13 months (average 6.67 ± 2.43 months). Inflammatory indicators including CRP, ESR, and WBC returned to normal levels within 2 to 8 weeks (average 4.48 ± 1.46 weeks). The clinical outcomes of patients were shown in Table 2. A typical case was shown in the Fig. 1.

**Table 2:** Summary of clinical outcomes of patients

Variables	
Operation time (min)	158.49 ± 23.3
Intraoperative blood loss (ml)	490.91 ± 298.3
External fixation time (month)	7.79 ± 2.81
Bone healing time (month)	6.67 ± 2.43
Time for inflammatory indicators returned to normal levels (week)	4.48 ± 1.46

Data shown as mean ± standard deviation. *P* < 0.05, significant difference

## Discussion

At present, there is still no consensus on the treatment of infection after intramedullary nail fixation. The choice of treatment plans depends on the doctor’s personal experience mostly. Some scholars advocated retaining intramedullary nails, and believe that intramedullary nails can provide sufficient stability for the fractured end. The stability of the fractured end plays a very important role in fracture healing. Therefore, they advocated performing local debridement to reduce the degree of infection and waiting for the fracture to heal. Turn the infection associated with fractures into separate infection. Compared with fracture healing, other scholars believed that the control of infection should be given priority. They advocated to remove intramedullary nails as soon as possible, perform local debridement and take corresponding measures to control infection. About infection after intramedullary nail fixation, we believe that the first purpose of treatment is to control the infection. The repair of bone defects is secondary. Because if the infection recurs, the treatment of the bone defect is meaningless. If the infection is controlled, the infectious bone defect can be converted into a single bone defect, which will reduce the difficulty of treatment greatly.

In the process of intramedullary nail fixation, the medullary cavity needs to be reamed, which will cause temporary damage to the blood supply in the medullary cavity. When the peripheral blood supply of the periosteum is normal, the blood supply inside the medullary cavity will be restored within 4–6 weeks [16]. The early infection of intramedullary nail fixation is manifested as a local infection, but the bacteria will spread in the entire medullary cavity along the intramedullary nail quiklly [17]. Due to the destruction of the blood supply in the medullary cavity and the presence of metal foreign bodies, bacterial biofilms will form quickly in the medullary cavity [18, 19]. In this case, the bacteria can survive under antibiotics and the autoimmune system, causing repeated infections. The treatment of infection after intramedullary nails needs to solve the situation of infection and instability of the fracture site. It is difficult to control intramedullary infection under the circumstance of local debridement. Therefore, it is extremely difficult to control the infection under the premise of retaining the internal fixation in the early stage. It was reported in the literature that the success rate of this method is only 33% [20]. For late infection, the presence of bacteria often affects fracture healing and causes infectious bone nonunion. The existence of internal fixation is not conducive to infection control, and the stability of the fracture site is a necessary condition to control infection and promote fracture healing [20]. Therefore, in this study, both early and late infections were treated with removing intramedullary nails, local debridement, topical antibiotics and external fixation.

Local application of antibiotics is very important in the control of bone infections [21]. Antibiotic irrigation and antibiotic bone cement implantation were used in the past as the usual methods. Some scholars have compared the clinical efficacy of antibiotic irrigation and antibiotic-loaded calcium sulfate in the treatment of chronic osteomyelitis, and found that the recurrence rate of infection and the probability of drainage tube obstruction were higher in this method [11]. Some scholars reviewed the clinical efficacy of the Ilizarov technique combined with antibiotic irrigation in the treatment of chronic osteomyelitis, and proved that antibiotic irrigation was still a good way to control infection on the basis of thorough debridement, but it would prolong the hospital stays and increase the economic burden of the patients significantly [22]. In order to solve this problem, the antibiotic delivery system emerged. In the 1970s, Professor Buchholz was the first person to apply bone cement mixed with antibiotics to revision surgery after hip arthroplasty, and found that the infection control rate can be increased to 90% without systemic antibiotics [23]. Professor Walenkamp reported 100 cases of chronic osteomyelitis treated with antibiotic bone cement beads. During the average follow-up period of 5 years, the infection control rate reached to 92% [24]. In order to provide local stability, some scholars used bone cement rods mixed with antibiotics to treat infectious bone nonunions. They believed that bone cement rods can integrate closely with the medullary cavity and eliminate dead spaces, which were beneficial to inflammation elimination and fracture healing [25]. However, antibiotic bone cement also has its own shortcomings, including: difficult placement in the medullary cavity, requirement for second operation to remove and unstable antibiotic release [3].

In this study, antibiotic delivery system we used is calcium sulfate, which has both tissue compatibility and osteoconductivity. Calcium sulfate is a biological material that can be degraded in the human body. The antibiotic loaded with calcium sulfate could reach the release peak within 6 ~ 24 h and maintain the antibacterial concentration for 6 ~ 8 weeks [26]. Moreover, the porous structure of calcium sulfate and method of layer-by-layer degradation can make the antibiotic to penetrate sufficiently, and avoid becoming a carrier for bacterial colonization in the late stage [27]. Some scholars have found that calcium sulfate can exhibits a microstructure similar to human cancellous bone, the formation of trabecular bone can be observed under light microscope after being absorbed in the human body [28]. Due to these advantages, calcium sulfate loaded with antibiotics has become more and more widely used in bone infections. Antibiotic artificial bone can not only release antibiotics slowly to achieve the purpose of continuous antibacterial, but also fill the bone defect. The later degradation process is also the process of new bone formation, which creates an opportunity for the repair of bone defects and accelerates the healing of the fracture site. In this study, fourteen patients received vancomycin calcium sulfate implantation, not only the infection was controlled and the fractures healed well without secondary autologous bone grafting.

The use of antibiotic artificial bone and external fixation to treat infection after intramedullary nail fixation requires the following considerations: (1) Thorough debridement has always played an important role in the treatment of bone infections. Reaming and debridement of the entire bone is required. The distal window should be opened to facilitate the discharge of inflammatory tissues and debris before reaming. (2) If the medullary cavity is closed due to bone filling, the bone window should be performed locally to remove bone tissue. (3) Vancomycin and gentamicin are the first choice for antibiotics topical application due to extensive antibacterial spectrum, thermal stability and low sensitization [29]. (4) In this study, 16 cases of Staphylococcus aureus, 4 cases of Pseudomonas aeruginosa, 1 case of Enterobacter cloacae, 5 cases of negative. Therefore, both vancomycin and gentamicin were added to calcium sulfate to ensure the efficacy. (5) Water will be produced during the degradation of calcium sulfate, which will cause large local drainage. So it is necessary to change the dressing regularly and take care of the wound [30]. In this study, one patient received vacuum sealing drainage (VSD) due to persistent postoperative exudation, another 5 patients controlled the continuous exudation after continuous dressing changes and wound care.

This study also has some limitations: (1) First of all, we did not set up a control group, and did not compare the efficacy of this method with the results of other methods. (2) Second, this is a retrospective study, which may be bias in the patients included in the study and the final results. (3) Third, the number of patients included in this study is small, so it is necessary to introduce a larger sample of randomized controlled trials to enhance the conviction of the conclusions.

## Conclusion

The available evidences show that antibiotic artificial bone implantation and external fixation is an effective method for the treatment of infection after intramedullary nail fixation.

## Supplementary Information

**Additional file 1.**

## Data Availability

The datasets generated and/or analysed during the current study are not publicly available due to limitations of ethical approval involving the patient data and anonymity but are available from the corresponding author on reasonable request**.**
