# Supplementary figures and images for: Antibiotic artificial bone implantation and external fixation for the treatment of infection after intramedullary nail fixation: a retrospective study of 33 cases
Source: BMC Musculoskelet Disord. 2022 Mar 5;23:209. doi: 10.1186/s12891-022-05161-8 (PMC8897969; doi:10.1186/s12891-022-05161-8)

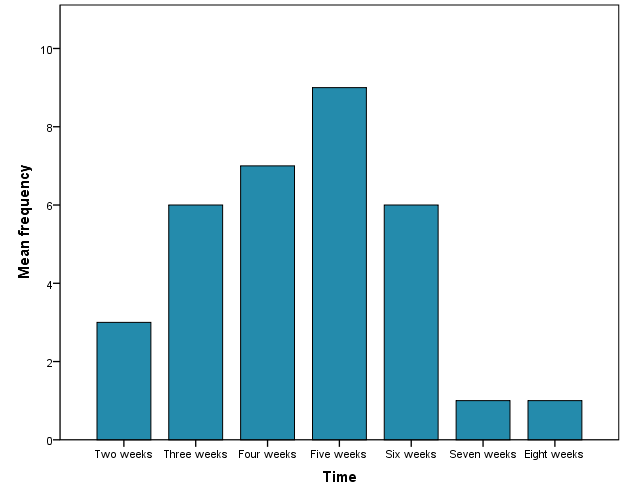


Time for inflammatory indicators returned to normal levels

Supplement: Supplementary file 1 — Additional file 1. [file 12891_2022_5161_MOESM1_ESM.docx]
